# Pathophysiological Responses of *Pinna nobilis* Individuals Enlightens the Etiology of Mass Mortality Situation in the Mediterranean Populations

**DOI:** 10.3390/cells10112838

**Published:** 2021-10-22

**Authors:** Athanasios Lattos, Konstantinos Feidantsis, Ioannis Georgoulis, Ioannis A. Giantsis, Dimitrios Karagiannis, John A. Theodorou, Alexandra Staikou, Basile Michaelidis

**Affiliations:** 1 Laboratory of Animal Physiology, Department of Zoology, School of Biology, Aristotle University of Thessaloniki, 54124 Thessaloniki, Greece; lattosad@bio.auth.gr (A.L.); kfeidant@bio.auth.gr (K.F.); georgoim@bio.auth.gr (I.G.); 2 Department of Animal Science, Faculty of Agricultural Sciences, University of Western Macedonia, 53100 Florina, Greece; igiantsis@uowm.gr; 3 National Reference Laboratory for Mollusc Diseases, Ministry of Rural Development and Food, 54627 Thessaloniki, Greece; vetaquapath@gmail.com; 4 Department of Animal Production Fisheries & Aquaculture, University of Patras, 26504 Mesolonghi, Greece; jtheo@upatras.gr; 5 Laboratory of Zoology, Department of Zoology, School of Biology, Aristotle University of Thessaloniki, 54124 Thessaloniki, Greece; astaikou@bio.auth.gr

**Keywords:** *Pinna nobilis*, mortality, pathogens, *Haplosporidium pinnae*, *Mycobacterium* sp., *Vibrio* spp., physiology, autophagy, cellular death, inflammation

## Abstract

Due to the rapid decrease of *Pinna nobilis* populations during the previous decades, this bivalve species, endemic in the Mediterranean Sea, is characterized as ‘critically endangered’. In addition to human pressures, various pathogen infections have resulted in extended reduction, even population extinction. While *Haplosporidium pinnae* is characterized as one of the major causative agents, mass mortalities have also been attributed to *Mycobacterium* sp. and *Vibrio* spp. Due to limited knowledge concerning the physiological response of infected *P. nobilis* specimens against various pathogens, this study’s aim was to investigate to pathophysiological response of *P. nobilis* individuals, originating from mortality events in the Thermaikos Gulf and Lesvos and Limnos islands (Greece), and their correlation to different potential pathogens detected in the diseased animals. In isolated tissues, several cellular stress indicators of the heat shock and immune response, apoptosis and autophagy, were examined. Despite the complexity and limitations in the study of *P. nobilis* mortality events, the present investigation demonstrates the cumulative negative effect of co-infection additionally with *H. pinnae* in comparison to the non-presence of haplosporidian parasite. In addition, impacts of global climate change affecting physiological performance and immune responses result in more vulnerable populations in infectious diseases, a phenomenon which may intensify in the future.

## 1. Introduction

The endemic sea bivalve species *Pinna nobilis* (Linnaeus, 1758) is one of the largest bivalve species in Mediterranean marine ecosystems, reaching up to 120 cm shell length and inhabiting coastal areas with *Posidonia oceanica* (Delile, 1813) or *Cymodocea nodosa* (Ucria) (Ascherson, 1870) meadows at depths of 0.5–60 m [1,2]. *P. nobilis* is characterized by its fast growth rate alongside other pinnids, with a recorded lifespan of about 20 years [3]. Due to the rapid decrease of *P.nobilis* populations due to its mass mortalities during the previous decades, this species is included in the Annex II Barcelona Convention (1992) and Annex IV of the EU habitats directive (2007), and it has been characterized as ‘critically endangered’ by the IUCN red list for threatened species [4,5]. Despite its endangered status, *P. nobilis* populations are highly threatened by human activities, such as illegal fishing for commercial and consumption purposes [6,7]. However, significant mortalities, even extinction in some cases, of *P. nobilis* within its natural habitats are mostly due to various pathogen infections [8]. While records of this phenomenon began in 2016, it continues to devastate the populations of fan mussels to the present day [9]. The newly described *Haplosporidium pinnae* is the most probable cause of this ecological risk [10].

The pathogen *Haplosporidium pinnae* was firstly described in Alicante (Spain) and the Balearic Islands (Spain), hosting *P. nobilis* specimens in locations that suffered heavy losses. Therefore, *H. pinnae* has been characterized as one of the major causative agents due to its presence in moribund and dead animals [9,11]. *H. pinnae* infection and haplosporidiosis continues to infect fan mussel populations in several Mediterranean marine areas (e.g., the Adriatic Sea, Ionian Sea, Aegean Sea, Tunisia, and Cyprus) [4,12,13,14,15,16], resulting in mass mortalities of these species [9,11,17]. Except for *H. pinnae*, *Mycobacterium* sp. have been detected in fan mussel specimens along the Italian (Campania and Sicily) and French coasts (Corsica), causing excessive histopathological lesions, acute immune responses, and large-scale mortalities [18]. Mycobacteriosis was also detected in fan mussel populations along the Greek coastline with similar consequences [4]. In addition to all the aforementioned potential pathogenic threats, opportunistic *Vibrio* spp. belonging to the Splendidus and Mediterranei clades were detected during mortality events in *P. nobilis* populations. Experimental laboratory conditions detecting the infection of *P. nobilis* by *Vibrio* spp have transformed the phenomenon of mortalities into a complicated matter [8,19,20,21,22]. Investigation of *P. nobilis* mortalities continued and more potential threats were implicated in the devastation of its natural populations [23,24].

However, little is known regarding the pathophysiological consequences caused, alone or synergistically, by each microorganism detected in *P. nobilis* and even less regarding the impacts on its physiological performance. Overall, immune responses of bivalves to the presence of pathogens rely on their innate immune system due to their inability to acquire immune responses and thus exhibit a stronger reaction in future encounters [25]. During the invasion of potential pathogens, the innate immune system of bivalves exhibits a strong response through pattern recognition receptors and triggers the downstream immune signaling pathways by the recognition of pathogen-associated molecular patterns [25,26,27]. Under the prism of pathogen infections, host cell death constitutes an intrinsic immune defense process for both invertebrates and vertebrates [28,29]. Apoptosis or programmed cell death can be triggered by external and internal factors [30]. The extrinsic pathway is activated upon stimulation of specific receptors for cell death, with the activation of Caspases being the final stage of the process [29]. The intrinsic pathway is activated through the release of signals by mitochondria and finally the activation of apoptotic proteins (Bcl-2 etc.) [29]. On the other hand, autophagy is a natural and fundamental mechanism that contributes to the management of intracellular biomass by self-digestion of cytoplasmatic components that range in complexity and size from individual proteins to whole organelles [31,32,33]. In addition to the aforementioned pathways, autophagy is also involved in cell homeostasis and other cellular processes, including adjustment to starvation, intracellular pathogen elimination, and cell death [34,35]. There are three distinct types of autophagy: microautophagy, macroautophagy, and chaperone-mediated autophagy [33]. All three types of autophagy are necessary processes for nutrient recycling in organisms; however, macroautophagy is the best studied [36]. Macroautophagy is involved in the isolation of cytoplasmic components within double-membrane vesicles, known as autophagosomes, and then the vesicles fuse with the lysosome in order to begin the degradation of cytoplasm [37]. Similarly, ubiquitination is the process for the degradation and removal of harmful proteins via the proteasome and also affects the retention of the paternal mtDNA in male bivalves [38,39]. However, cell protection is supported by heat shock proteins (Hsps) [40], which are responsible for the assembly and folding as well as translocation of proteins and also assisting in the degradation of structural aberrant proteins [40], thereby assisting in disease resistance and environmental changes in all aquatic organisms. Hsps dynamically assist in the stimulation of the secretion of inflammatory cytokines such as nitric oxide synthase, tumor necrosis factor-a and interleukins IL-1β and IL-6 [41].

Keeping in mind the lack of complete knowledge concerning the physiological response of infected *P. nobilis* specimens against various pathogens, the objectives of this study are the investigation of the pathophysiological responses of *P. nobilis* individuals originating from mortality events, as well their correlation to the different potential pathogens detected in the diseased animals. In order to assess its pathophysiological response, cell indicators of heat shock (Hsp70 and Hsp90) and immune response (Il-6 and TNFα) as well as autophagic (ubiquitin, LC3, and SQSTM1/p62) and apoptotic (caspases) indicators were examined through both Western blot and dot blot analysis, as well as with immunohistochemistry (IHC).

## 2. Materials and Methods

### 2.1. Animal and Tissue Sampling

The digestive gland, gills, and mantle obtained from specimens originating from mass mortality events, as described in Lattos et al. [4] (Table 1), were utilized for the investigation of the pathophysiology of *Pinna nobilis*.

**Figure 1:**
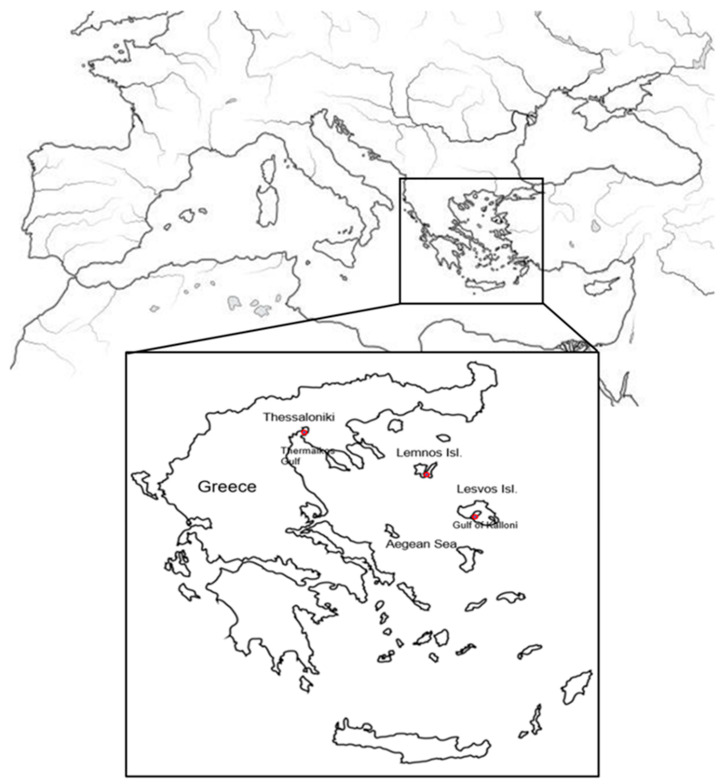
Origin of specimens used for the research.

Specimens used for the microbiome analysis [19] were also used for the needs of this study. Among the specimens used for this study, two of them originated from Kalloni Gulf (Lesvos island) (Kal10, Kal12), three of them originated from Thermaikos Gulf (Thessaloniki) (Ther01, Ther02, Ther03), whereas the last specimen originated from Limnos island (Lim01) (Table 1). All the above areas exhibited a similar level of mass mortalities. However, contrary to Lesvos island, where all specimens were dead, moribund and alive specimens were found in Limnos island and in Thermaikos Gulf, respectively. The origin of specimen origin has been described in detail in Lattos et al. [4] (Figure 1). Specimens from the Thermaikos Gulf have been collected during May 2019 (17 °C) in *C. nodosa* meadows of 5–8 m depth, from Kalloni Gulf (Lesvos island) during January 2019 (13 °C) in *P. oceanica* meadows of 5–6 m depth, and from Limnos island during June 2019 (16 °C) in *P. oceanica* meadows of 3–5 m depth.

Since *Pinna nobilis* is considered a highly endangered species, special permission was requested and received from local authorities in order to collect specimens (МЕЕ/GDDDP89926/1117).

### 2.2. Histopathological Procedures

Histopathological processes presented herein are described in detail in Lattos et al. [4]. In brief, tissue parts were fixed immediately for 48 h in the Davidson solution, and after dehydration through graded alcohols, samples were embedded in paraffin wax and sectioned at 4–5 μm using a rotary microtome. After the samples were stained with Hematoxylin and Eosin, Ziehl–Neelsen staining was also applied to the specimens in order to examine the presence of mycobacteria parasites.

### 2.3. Immunohistochemistry Procedure for Protein Localization

Embedded tissue paraffin blocks, obtained from specimens that originated in Greek mortality events, were used for the protein localization in sampled tissue [4]. Embedded tissues in paraffin wax were sectioned at 4–5 μm using a rotary microtome and mounted in positive charged slides. Sections were left at room temperature overnight for optimal drying. Deperaffinization was performed with immersion of mounted sections in Dewax and Hier Buffer H (Thermo Fisher Scientific, Waltham, MA, USA), and staining was held according to manufacturer’s protocol with UltraVisionQuanto HRP Detection System (Thermo Fisher Scientific, USA). The antibodies used were monoclonal mouse anti-hsp90 (H1775, Sigma, Germany), anti-IL-6 (CSB-PA06757A0Rb, Cusabio, Houston, TX, USA), anti-TNFα (CSB-PA07427A0Rb, Cusabio, USA), anti-cleaved caspase antibody (Cat. No.8698 Cell Signalling, Oxford, UK), polyclonal anti-ubiquitin rabbit antibody (Cat. No. 3936, Cell Signalling, UK), and monoclonal rabbit anti-LC3B (3868, Cell Signaling, UK).

### 2.4. SDS-PAGE/Immunoblot and Dot Blot Analysis

#### 2.4.1. Preparation of Tissue Samples

Tissue samples were homogenized (1/3 *w*/*v*) in cold lysis buffer (20 mM β-glycerophosphate, 50 mM NaF, 2 mM EDTA, 20 mM Hepes, 0.2 mM Na_3_VO_4_, 10 mM benzamidine, pH 7, 200 μM leupeptin, 10 μΜ trans-epoxy succinyl-L-leucylamido-(4-guanidino)butane, 5 mM dithiotheitol, 300 μΜ phenyl methyl sulfonyl fluoride (PMSF), 50 μg mL^−1^ pepstatin, and 1% *v*/*v* Triton X-100). After a 30 min extraction on ice, samples were centrifuged (10,000× *g*, 10 min, 4 °C) and the supernatants were boiled (3/1 *v*/*v*) with sample buffer (330 mM Tris-HCl, 13% *v*/*v* glycerol, 133 mM DTT, 10% *w*/*v* SDS, 0.2% *w*/*v* bromophenol blue). Protein concentrations were determined using the BioRad protein assay.

#### 2.4.2. SDS-PAGE/Immunoblot

Indicators of the autophagic and apoptotic pathways were determined in mantle and PAM samples according to well-established protocols for SDS-PAGE/immunoblot analysis. Specifically, equivalent amounts of proteins (80 μg) were separated on 10% (*w*/*v*) acrylamide and 0.275% (*w*/*v*) bisacrylamide slab gels, and transferred electrophoretically onto nitrocellulose membranes (0.45 μm, Schleicher & Schuell, Keene N. H. 03431, USA). Non-specific binding sites on the membranes were blocked with 5% (*w*/*v*) non-fat milk in TBST (20 mM Tris-HCl, pH 7.5, 137 mM NaCl, 0.1% (*v*/*v*) Tween 20) for 30 min at room temperature. Nitrocellulose membranes resulting from the above procedure were subjected to overnight incubation with monoclonal mouse anti-hsp70 (H5147, Sigma, Germany), monoclonal mouse anti-hsp90 (H1775, Sigma, Germany), anti-IL-6 (CSB-PA06757A0Rb, Cusabio, USA), anti-TNFα (CSB-PA07427A0Rb, Cusabio, USA), and polyclonal rabbit anti-p62/SQSTM1 (5114, Cell Signaling, UK). Quality transfer and protein loading were assured by Ponceau stain and actin (anti-β actin 3700, Cell Signaling, UK) (data not shown). Antibodies were diluted as recommended by the manufacturer’s guidelines. After washing in TBST (3 periods, 5 min each time), the blots were incubated with horseradish peroxidase-linked secondary antibodies, washed again in TBST (3 periods, 5 min each time), and the bands were detected using enhanced chemiluminescence (Chemicon) with exposure to Fuji Medical X-ray films. Films were quantified by laser-scanning densitometry (GelPro Analyzer Software, GraphPad, San Diego, CA, USA).

#### 2.4.3. Dot Blot Analysis

Cleaved caspases and ubiquitin conjugate levels were determined in mantle and PAM samples with the employment of a dot blot apparatus. Specifically, samples were diluted to a concentration of 5 μg mL^−1^ in a saline solution (150 mM NaCl); 100 μL volumes were loaded onto a pre-soaked nitrocellulose membrane (0.45 μm) in a dot blot vacuum apparatus (BioRad), and gravity-fed through the membrane. The membrane was blocked with 5% (*w*/*v*) non-fat milk in TBST (20 mM Tris-HCl, pH 7.5, 137 mM NaCl, 0.1% (*v*/*v*) Tween 20) for 30 min at room temperature. The resulting nitrocellulose membrane was subjected to overnight incubation with anti-cleaved caspase antibody (Cat. No.8698 Cell Signaling, UK) and polyclonal anti-ubiquitin rabbit antibody (Cat. No. 3936, Cell Signaling, UK). Antibodies were diluted as recommended by the manufacturer’s guidelines. After washing in TBST (3 periods, 5 min each time), the dots were incubated with horseradish peroxidase-linked secondary antibodies, washed again in TBST (3 periods, 5 min each time), and the dots were detected using enhanced chemiluminescence (Chemicon) with exposure to Fuji Medical X-ray films. Films were quantified by laser-scanning densitometry (GelPro Analyzer Software, GraphPad, USA).

### 2.5. Statistical Analysis

One-way analysis of variance (ANOVA) (GraphPad Instat 3.0, USA) followed by Bonferroni post hoc analysis was employed to test for significance at *p* < 0.05 (5%) level between all experimental groups examined herein. Since normality tests have little power to test the homogeneity of data for small sample sizes (as the ones described herein—n = 3 technical replicates), Friedman’s non-parametric test and Dunn’s post-test were applied. Moreover, correlation analysis between the biochemical indicators examined in the present study was conducted in order to establish significant relations.

## 3. Results

**Figure 2:**
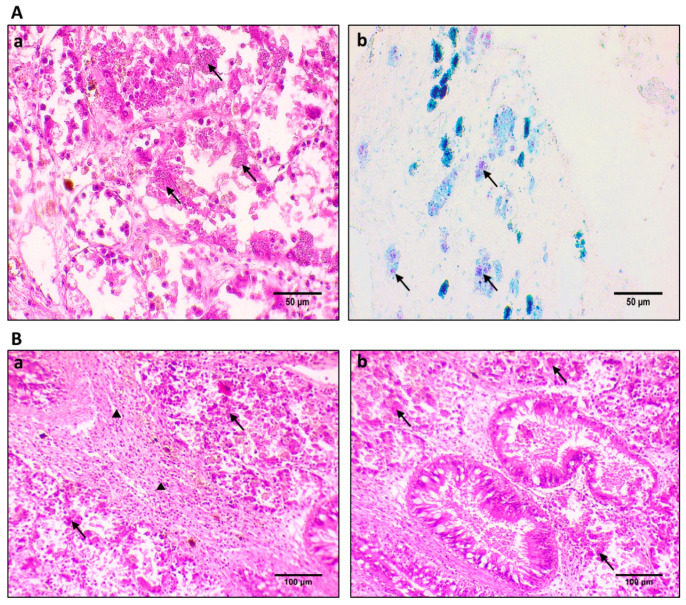
Histological sections of *P. nobilis*, showing sporulation stages of *H. pinnae* and *Mycobacterium* sp. in the digestive gland of infected animals. (**Aa**) Sporulation stages of *H. pinnae* (arrows). (**Ab**) Gram-positive acid-fast mycobacteria (arrows) on sections stained with ZN staining. (**Ba**) Diffuse type inflammation in digestive gland of infected specimens with *H. pinnae* (arrows) and heavy haemocyte infiltration in the surrounding connective tissue (triangle). (**Bb**) Display of heavy lesions in the digestive gland of infected animals with the presence of *H. pinnae* (arrows).

*Haplosporidium pinnae* was histologically detected in four out of the six samples used for this research (Figure 2(Aa,Ba,Bb)), while *Mycobacterium* sp. was also histologically detected in all samples used in this research (Figure 2(Ab)). All aforementioned microorganisms were molecularly identified, and all novel sequences were deposited in the Genbank database in our previous study [4]. Individuals originating from the Thermaikos Gulf showed a better health condition regarding macroscopical results, while individuals originating from the Kalloni Gulf (Lesvos Island) and Limnos Island exhibited macroscopical signs attributed to heavy infections [4]. *H. pinnae* was detected in all its stages in the digestive gland of the infected individuals. Moreover, *H. pinnae* detection was accompanied with heavy lesions in the connective tissue of the digestive gland of each individual, while high-density degenerative process in the epithelial tissue was also observed (Figure 2(Aa,Ba,Bb)). Along with the aforementioned processes, heavy haemocytic infiltration was detected in all tissues; in the connective tissue of the digestive gland, it was detected in higher density (Figure 2(Ba)). Brown cell formation was also present in the digestive gland as a sign of the pathogen invasion in the individuals (Figure 2(Ba)). Regarding *Mycobacterium* sp., the existence of purple rod-shaped, acid-fast, Gram-positive bacilli was presented, filling the immune cell constituents in all individuals examined in this research.

Regarding the detection of the proteins examined in this research, Western blotting, dot blotting, and IHC techniques were employed to quantify and localize the expression between the individuals infected by both parasites and the individuals infected only by *Mycobacterium* sp. IHC results showed different expression patterns in each protein in the digestive gland tissue examined for this research. Labeling of primary antibodies was observed as brown precipitates (indicated by black arrows), the product of DAB (3,3′-Diaminobenzidine) chromogen. In general, no differences were observed in the expression and localization of the proteins observed as a result of IHC between the individuals infected only with *Mycobacterium* sp. and the individuals infected by both pathogens.

**Figure 3:**
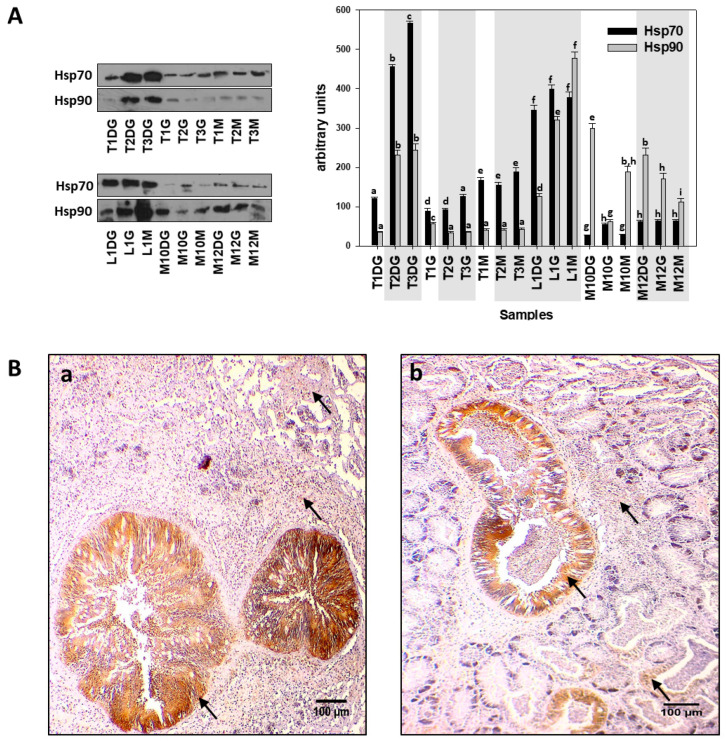
(**A**). Hsp70 and Hsp90 levels in the digestive gland (DG), gills (G), and mantle (M) of *Pinna nobilis* specimens from the Kalloni Gulf (Lesvos Island) (M10, M12), from Thermaikos gulf (Thessaloniki) (T01, T02, T03), and from Limnos Island (L1). All specimens were infected with mycobacteria and vibrios, while grey shaded areas represent specimens additionally infected with the haplosporidian parasite. Tissue extracts from all groups were immunoblotted for Hsp70 and Hsp90. Blots were quantified using scanning densitometry. Representative blots are shown. Lower-case letters indicate statistically significant differences (*p* < 0.05) between samples. (**B**) (**a**,**b**) Immunohistochemical detection (arrows) of Hsp90 in the digestive tubule and in the connective tissue of the digestive gland of non-infected (**a**) and infected (**b**) specimens from *H. pinnae*.

Hsp70 was localized, in both cases, mostly in the epithelial cell of the digestive gland and less on the connective tissue (Figure 3(Ba,Bb)). In contrast, Western blot analysis indicated that individuals additionally infected by *H. pinnae* exhibited higher levels of both Hsp70 and Hsp90 in all three examined tissues, compared to the non-*H. pinnae*-infected individuals, as depicted in grey shaded areas in Figure 3A.

**Figure 4:**
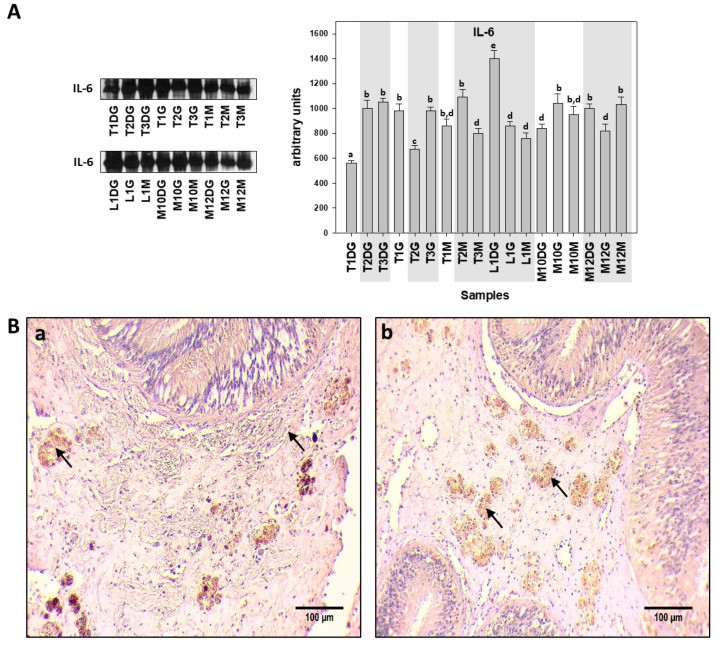
(**A**). Il-6 levels in the digestive gland (DG), gills (G), and mantle (M) of *Pinna nobilis* specimens from Kalloni Gulf (Lesvos Island) (M10, M12), Thermaikos Gulf (Thessaloniki) (T01, T02, T03), and Limnos Island (L1). All specimens were infected with mycobacteria and vibrios, while grey shaded areas represent specimens additionally infected with the haplosporidian parasite. Tissue extracts from all groups were immunoblotted for Il-6. Blots were quantified using scanning densitometry. Representative blots are shown. Lower-case letters indicate statistically significant differences (*p* < 0.05) between samples. (**B**). (**a**,**b**) Immunohistochemical detection (arrows) of Il-6 in the connective tissue of *P. nobilis* digestive gland of non-infected (**a**) and infected (**b**) specimens by *H. pinnae*. (in Ba, left arrow indicates concentrated, while right arrow indicates scattered Il-6 detection).

In contrast to the Hsp levels observed, analysis of Il-6 levels detected by Western blot did not reveal differences between the *H. pinnae* infected and non-*H. pinnae*-infected individuals (Figure 4A), except for the digestive gland (DG) of individuals from Thermaikos gulf, where Il-6 levels were higher in *H. pinnae* infected individuals. This pattern of non-significant differences between *H. pinnae* infected and non-*H. pinnae* infected individuals was also confirmed by IHC detection of Il-6, which was observed in both connective and epithelial tissue, regardless of the *H. pinnae* presence (Figure 4(Ba,Bb)).

**Figure 5:**
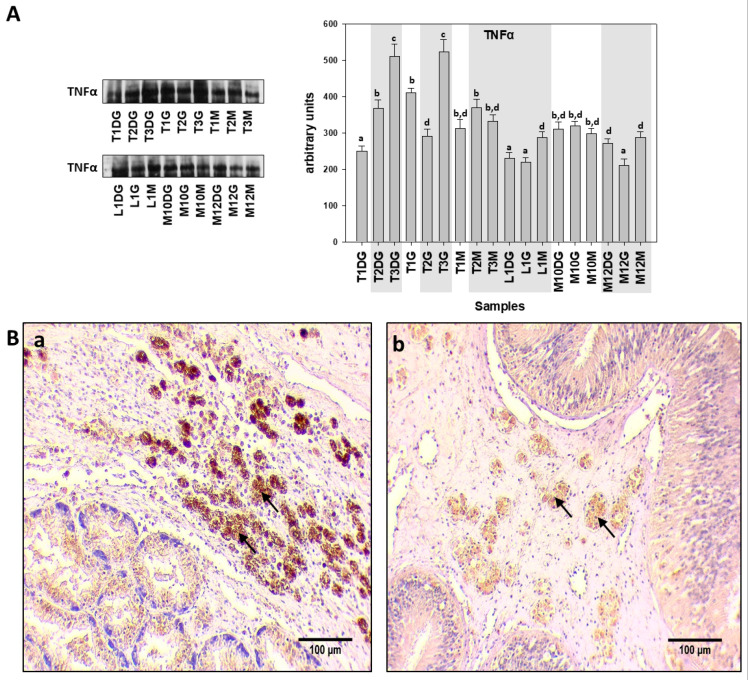
(**A**). TNFα levels in the digestive gland (DG), gills (G), and mantle (M) of *Pinna nobilis* specimens from Kalloni Gulf (Lesvos Island) (M10, M12), from Thermaikos Gulf (Thessaloniki) (T01, T02, T03), and from Limnos Island (L1). All specimens were infected with mycobacteria and vibrios, while grey shaded areas represent specimens additionally infected with the haplosporidian parasite. Tissue extracts from all groups were immunoblotted for TNFα. Blots were quantified using scanning densitometry. Representative blots are shown. Lower case letters indicate statistically significant differences (*p* < 0.05) between samples. (**B**). (**a**,**b**) Immunohistochemical detection (arrows) of TNFa, in the connective tissue of *P. nobilis* digestive gland of non-infected (**a**) and infected (**b**) specimens by *H. pinnae*.

A similar pattern to the Il-6 expression was observed concerning TNFa. Thus, TNFa levels detected by Western blot analysis revealed differences between the *H. pinnae* infected and non-*H. pinnae*-infected individuals only in the digestive gland (DG) of Thermaikos gulf individuals (Figure 5A). The pattern expression of the TNFa by IHC detection was similar to Il-6 as described above (Figure 5(Ba,Bb)).

**Figure 6:**
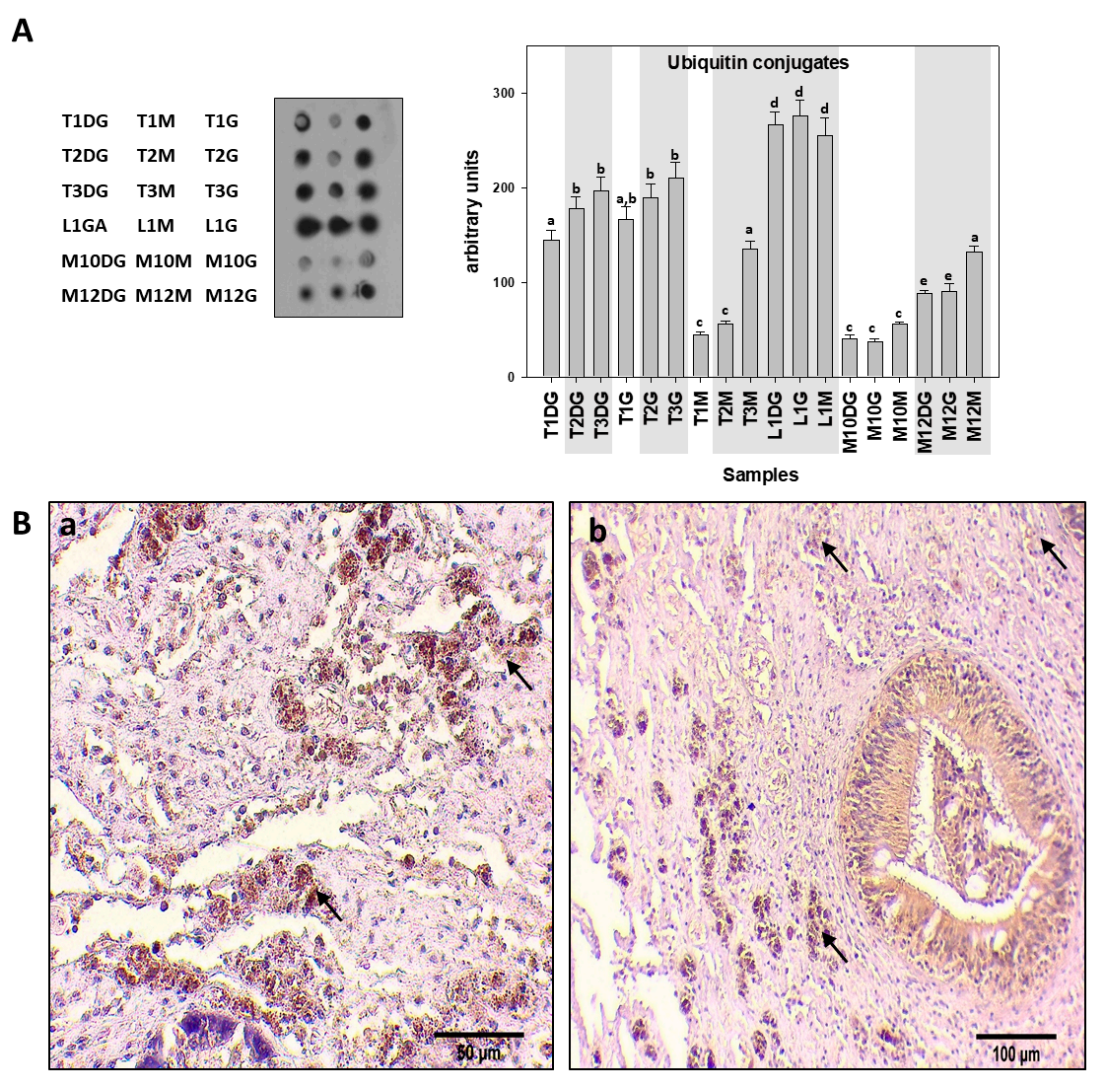
(**A**). Ubiquitin conjugates levels in the digestive gland (DG), gills (G), and mantle (M) of *Pinna nobilis* specimens from the Kalloni Gulf (Lesvos Island) (M10, M12), from Thermaikos gulf (Thessaloniki) (T01, T02, T03), and from Limnos Island (L1). All specimens were infected with mycobacterium and vibrio, while grey shaded areas represent specimens additionally infected with the haplosporidian parasite. Tissue extracts from all groups were immunoblotted for ubiquitin. Dots were quantified using scanning densitometry. Representative dots are shown. Lower-case letters indicate statistically significant differences (*p* < 0.05) between samples. (**B**). (**a**,**b**) Immunohistochemical detection of ubiquitin (arrows) in the connective tissue of the digestive gland of non-infected (**a**) and infected (**b**) specimens from *H. pinnae*.

The ubiquitin conjugate levels exhibited the same pattern as Hsp levels. Specifically, the ubiquitin conjugate levels, as detected by Western blot analysis, were higher in all three examined tissues of individuals infected additionally with *H. pinnae*, compared to the non-*H. pinnae* infected ones, as depicted in grey shaded areas in Figure 6A. However, IHC localization of ubiquitin was observed in the same density in both the connective and epithelial tissues in both cases (Figure 6(Ba,Bb)).

**Figure 7:**
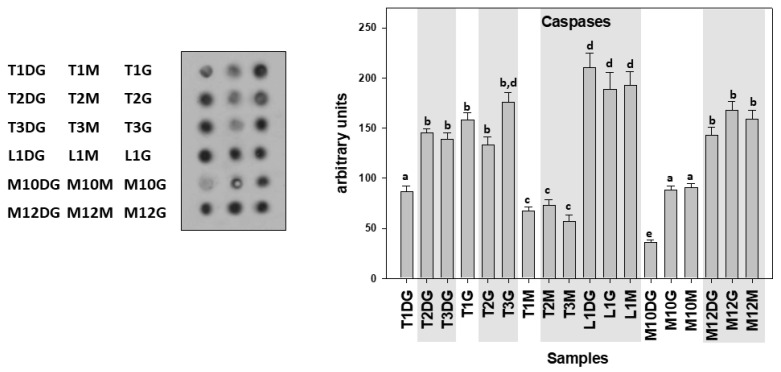
Cleaved caspases levels in the digestive gland (DG), gills (G), and mantle (M) of *Pinna nobilis* specimens from the Kalloni Gulf (Lesvos Island) (M10, M12), from the Thermaikos Gulf (Thessaloniki) (T01, T02, T03), and from Limnos Island (L1). All specimens were infected with mycobacterium and vibrio, while grey shaded areas represent specimens additionally infected with the haplosporidian parasite. Tissue extracts from all groups were immunoblotted for caspases. Dots were quantified using scanning densitometry. Representative dots are shown. Lower-case letters indicate statistically significant differences (*p* < 0.05) between samples.

Concerning cleaved caspases, its appears that although individuals infected by *H. pinnae* in Limnos (L) and Lesvos (M) islands exhibited higher levels compared to the non-infected ones, Thermaikos Gulf individuals exhibited a different pattern. Specifically, caspase levels exhibited differences between the *H. pinnae* infected and non-*H. pinnae*-infected individuals only in the digestive gland (DG) of Thermaikos Gulf individuals (Figure 7).

**Figure 8:**
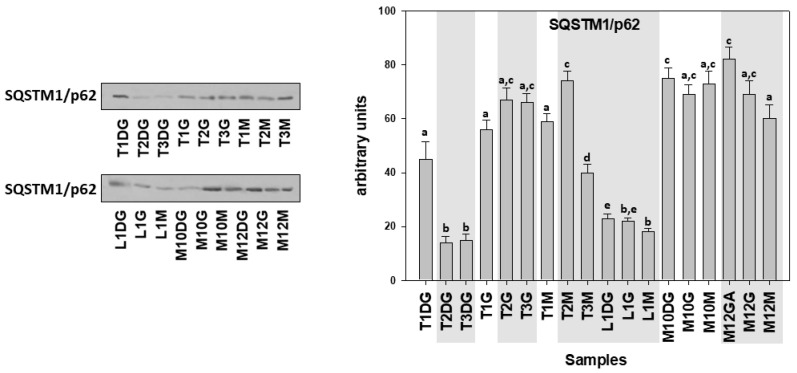
SQSTM1/p62 levels in the digestive gland (DG), gills (G), and mantle (M) of *Pinna nobilis* specimens from the Kalloni Gulf (Lesvos Island) (M10, M12), from the Thermaikos Gulf (Thessaloniki) (T01, T02, T03), and from Limnos Island (L1). All specimens were infected with mycobacterium and vibrio, while grey shaded areas represent specimens additionally infected with the haplosporidian parasite. Tissue extracts from all groups were immunoblotted for SQSTM1/p62. Blots were quantified using scanning densitometry. Representative blots are shown. Lower-case letters indicate statistically significant differences (*p* < 0.05) between samples.

The same pattern with cleaved caspases levels was observed concerning SQSTM1/p62 levels, as these levels were decreased in all three examined tissues of individuals infected by *H. pinnae* in Limnos (L) and in the digestive gland (DG) of the Thermaikos Gulf *H. pinnae* infected individuals (Figure 8).

**Figure 9:**
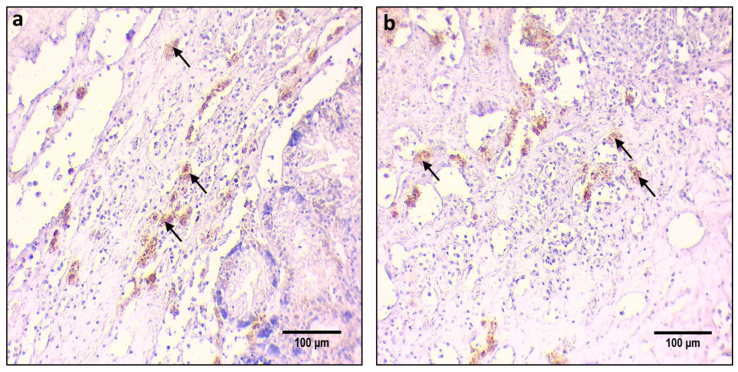
Immunohistochemical detection of the digestive gland tissue showing detected antibodies (arrows) of LC3B in the connective tissue of non-infected (**a**) and infected (**b**) from *H. pinnae* individuals.

Figure 9 depicts the IHC localization of LC3B. As it is shown, it is mostly observed on epithelial cells of both *H. pinnae* infected and non-infected cases.

Table 2 depicts the correlation between the examined biochemical indicators in the present study. The most significant correlation is found between Hsps, ubiquitin and the autophagic indicator SQSTM1/p62.

## 4. Discussion

Population reduction of fan mussel continuing along the Greek coastline has resulted in heavy population losses in both the Aegean and Ionian seas, even in 2021. Mortalities of *P. nobilis* in Greek territories have modified the distribution of the species. Subsequently, *P. nobilis* populations may be restricted to only deeper seabed topologies. The regeneration status in shallow waters is at low levels, reaching up to zero percent in many cases due to the extensive heat in the summer of 2021.

Disease aetiology of mass mortality events in *P. nobilis* species is considered a complex situation, involving many microorganisms in relation to rapid changes in abiotic factors due to ongoing climate change [21]. Infection by *H. pinnae* was adopted as a main causative agent in many cases without taking into consideration the pathobiome of the species [21]. Furthermore, a lack of healthy–uninfected populations and a limited number of alive individuals further jeopardise the accession to the pathogenesis of the microorganisms to the infected species. Additionally, the lack of individuals and the rapid decrease in all the populations of the Mediterranean Sea constitute an obstacle in order to correlate the effect of abiotic factors directly to the microorganisms detected in moribund specimens.

The results of the current research are in line with the results of Box et al. [42], who estimated for the first time *P. nobilis* physiological performance, after the comparison of infected and uninfected animals. Their study confirmed the theory of multifactorial mortalities with many pathogens, each of those having a cumulative negative effect on host physiology of the species. The results of their study demonstrated the reduction of antioxidant defenses in the presence of both *H. pinnae* and *Mycobacterium* sp., and the cumulative effect of both pathogens in comparison with both the uninfected and the individuals hosting only one of them. Herein, analysis of both Hsp70 and Hsp90 demonstrated a similar pattern in both cases. Individuals infected with all three pathogens exhibited higher levels of protein induction, indicating an increased cellular stress response in comparison with the individuals infected only with mycobacterium and vibrio. Higher protein induction in individuals with all three pathogens occurred even in the same sampling with the same environmental conditions. Keeping in mind that Hsps enable cells to adapt to various stresses, it is obvious that this cellular mechanism maintains normal cellular functions by counteracting misfolded cellular proteins [43]. This is consistent with the decreased levels of Hsps in vital tissues, such as the gills and mantle of the Thermaikos individuals, since the latter exhibited a better health condition regarding macroscopical results, compared to individuals collected from the Kalloni Gulf (Lesvos Island) and Limnos Island, which exhibited heavy macroscopical infection signs such as extended lesion sites [4]. Although the relation of Hsps’ induction with lesion formation has not been evidenced in marine organisms, it has been shown in mammalian animal models. Specifically, Hsp70 is strongly upregulated very early at lesion-prone sites in young apoE^−/−^ knockout mice aortas [44]. Pathogenic infection induces Hsp70 and Hsp90 in marine bivalves which are closely related to immune responses [45,46,47].

Although Hsps seem to play a pivotal role in the induction of pro-inflammatory cytokines’ production [48], the present study showed that the expression patterns of both Il-6 and TNF-α did not follow the Hsps’ pattern of induction. Specifically, Il-6 expression did not follow any pattern on infections, although it is considered to be an important cytokine in regulating immune responses and was allocated mostly in the connective tissue of the digestive gland tissue examined through IHC [49]. Additionally, TNF-α, which is produced to induce an inflammatory reaction [50], similarly to Il-6, did not demonstrated any significant difference between individuals infected additionally with *H. pinnae* and individuals with both pathogens. Moreover, the Il-6 and TNF-α levels did not exhibit any notable differences between the samplings through different seasons in the year, resulting in the fact that inflammatory responses are at high levels throughout the year. The latter shows that infected populations in both cases of infection present consistently high stress levels. Concerning the immunohistochemical localization of these two important cytokines in the digestive gland, they seemed to be scattered around the digestive gland without any limitation in their localization.

Ubiquitin conjugate levels presented the same expression pattern as Hsps mentioned before, as also seen in Table 2. This may be attributed to the fact that in eukaryotic cells, ubiquitin and certain ubiquitin-conjugating enzymes are Hsps that function in the rapid turnover of denatured proteins [51]. In the case of infection with both *H. pinnae* and *Mycobaterium* sp., the ubiquitination levels were significantly higher than in the case of infection only with the haplosporidan parasite, thereby strengthening the important role of ubiquitin in stress and immune responses [52].

Due to the fact that ubiquitin is a ubiquitously expressed protein that can be covalently connected to selected proteins, as well as tag proteins for proteasomal or lysosomal degradation, its connection to cell death pathways is indisputable [53]. Cell death pathways such as apoptosis and autophagy account for self-destructive processes through which excessive cellular damage and damaged organelles are abolished. Although the successful corporation between apoptosis and autophagy is a very complex process, the choice of which response is going to be followed depends on the stimulus potency [54]. Therefore, while due to several stimuli, the autophagic pathway may prevent cell death by eliminating apoptosis, other stimuli may trigger autophagy as an alternative cell death possibility [53]. Regarding apoptosis, caspases, which have an important role in cell death activation, their levels accord with the result pattern of Hsps and ubiquitin, confirming the importance they pose in pathogenetic stress conditions of the host [55]. Herein, the obtained results have not shown a similar pattern of expression for autophagy. Concerning autophagy, although due to unknown facts, LC3 was not detected through Western blot in the present study, the SQSTM1/p62 exhibited a tissue specific pattern of expression, indicating lower levels and therefore increased autophagy in individuals additionally infected with the haplosporidian parasite. The protein is itself degraded by autophagy and may serve to link ubiquitinated proteins to the autophagic machinery to enable their degradation in the lysosome [56]. Although the aforementioned biochemical pathways have also been studied in other aquatic organisms such as teleosts [57], the sequence of these events has not been examined, since the latter is beyond this study’s aim. Tissue-specific response in most of the different proteins examined in the present study has also been observed both in invertebrate and vertebrate marine organisms [58,59].

Conclusively, disease aetiology of the fan mussel, *P. nobilis* and the co-infection by several microorganisms, accompanied by the limited individuals left in Greek seas, result in devastating to the species population effects. Although water physicochemical parameters were different between examined areas, no correlation between these parameters and the mortality status of the populations could be concluded. This fact could be attributed to the general limitations regarding *P. nobilis* studies due to this species status. Despite the complexity and limitations in the study of *P. nobilis* mortality events, the results of this research demonstrate the additive negative effect of co-infection in comparison with the additional infection by the *H. pinnae*. Fan mussels infected by all the main referred pathogens exhibited lower physiological performance compared with Fan mussels hosting only *H. pinnae*. Histopathological lesions originating from previous research confirmed the negative effect on the hosts infected only by *Mycobacterium* sp. [4]. Co-infections of multiple pathogenic microorganisms can affect the progression and disease pathogenesis, the transmission of the disease among the same population and the clinical effects on the host [23]. In addition, impacts of global climate change, such as oceanic warming and acidification, affecting physiological performance and immune responses, result in more vulnerable to infectious diseases populations [60,61], a phenomenon which may intensify in the future. The latter is due to the rapid climate change and its effects, as they consist of a major stressor in all terrestrial and marine ecosystems and in their habitats [4]. The main impacts of global climate change are reportedly linked to phenomena such as decreased ocean productivity, changes in species habitat distribution, and the spread of pathogens [62]. In addition, changes in abiotic parameters can affect the physiological performance of the species, leading to lower immune responses [63,64]. Moreover, it should be mentioned that decreased physiological performance could have an impact on the affected immune responses, which may eventually lead to the uncontrollable growth of opportunistic microorganisms, and finally to the detriment of the host [65,66,67].

## Figures and Tables

**Table 1:** Data of the specimens used for the study.

Specimen ID	Shell Length, Height, Width (cm)	Weight (gr)	Tissue	Haplosporidiosis	Mycobacteriosis	Vibriosis
Ther01			DG		+	+
	52.2, 14.2, 6.6	1482.26	G			
			M			
Ther02			DG	+	+	+
	56.5, 13.5, 6.3	1543.72	G			
			M			
Ther03			DG	+	+	+
	68.6, 18.4, 7.3	3080.42	G			
			M			
Lim01			DG	+	+	+
	22.3, 8.1, 2.5	160.2	G			
			M			
Kal10			DG		+	+
	34.2, 12.8, 4.3	298.26	G			
			M			
Kal12			DG	+	+	+
	29.3, 11.4, 3.5	208.75	G			
			M			

Kal10, Kal12: Kalloni Gulf—Lesvos island, Ther01, Ther02, Ther03: Thermaikos Gulf—Thessaloniki, Lim01: Limnos island. DG: Digestive gland, G: Gills, M: Mantle.

**Table 2:** Correlation analysis between biochemical parameters examined with Western blot and dot blot analysis.

	Hsp70	Hsp90	Il-6	TNF-α	Ubiquitin	Caspases	SQSTM1/p62
**Hsp70**	1						
**Hsp90**	0.463	1					
**Il-6**	0.228	−0.042	1				
**TNF-α**	0.228	−0.197	0.211	1			
**Ubiquitin**	0.673	0.318	0.085	0.049	1		
**Caspases**	0.421	0.332	0.314	−0.065	0.787	1	
**SQSTM1/62**	−0.921	−0.425	−0.102	−0.063	−0.754	−0.447	1

## Data Availability

The data presented in this study are available on request from the corresponding author. The data are not publicly available due to local authorities’ privacy restrictions.
